# TipQuant: a robust algorithm for quantitative analysis of spatiotemporally dynamic activities in tip‐growing cells

**DOI:** 10.1111/nph.71474

**Published:** 2026-08-20

**Authors:** Jingzhe Guo, Julian Le Gouic, Rémi Rosenthal, Ailing Zou, Xiang Zhou, Nicolas Brunel, Zhenbiao Yang, Xinping Cui

**Affiliations:** ^1^ Institute for Integrative Genome Biology and Department of Botany and Plant Sciences University of California Riverside CA 92521 USA; ^2^ Capgemini Invent 92130 Issy‐les‐Moulineaux France; ^3^ Quantmetry 52, rue d'Anjou 75008 Paris France; ^4^ Metabolomics Research Center, Haixia Institute of Science and Technology Fujian Agriculture and Forestry University Fuzhou 350002 China; ^5^ State Key Laboratory of Quantitative Synthetic Biology Shenzhen Institute of Synthetic Biology, Shenzhen Institutes of Advanced Technology, Chinese Academy of Sciences Shenzhen 518055 China; ^6^ ENSIIE & Laboratoire de Mathématiques et Modélisation d'Evry Université Paris Saclay 91025 Evry France; ^7^ Institute of Emerging Agricultural Technology Shenzhen University of Advanced Technology Shenzhen 518107 Guangdong China; ^8^ Faculty of Synthetic Biology Shenzhen University of Advanced Technology Shenzhen 518107 Guangdong China; ^9^ Institute for Integrative Genome Biology and Department of Statistics University of California Riverside CA 92521 USA

**Keywords:** cell polarity, quantitative analysis, robust algorithm, spatiotemporal dynamics, tip growth

## Abstract

- Cell polarity and tip growth rely on the dynamic spatial organization of signaling and structural components. Quantitative characterization of these spatiotemporal dynamics is critical for understanding polarized cell growth, yet manual quantification is labor‐intensive and existing computational tools often lack the flexibility and robustness needed to analyze molecular and structural dynamics in tip‐growing cells.
- Tip Quantification (TipQuant) identifies the cell apex by detecting the site of maximum expansion and automatically quantifies fluorescence distribution along the plasma membrane and within the apical cytoplasm from live‐cell imaging data, enabling analysis of the spatiotemporal dynamics of molecular and structural components in tip‐growing cells.
- TipQuant accurately identified cell apices and quantified the spatiotemporal behavior of fluorescently labeled proteins and cellular structures in *Arabidopsis thaliana* pollen tubes and *Fusarium graminearum* hyphae, reproducing manual measurements while reducing user bias and improving efficiency, consistency, and analytical flexibility. The tool also revealed a strong positive correlation between rho‐like GTPase from plants activity and apical Ca^2+^ influx in Arabidopsis pollen tubes, demonstrating its utility for analyzing dynamic cellular processes.
- TipQuant is a robust analytical tool for quantifying spatiotemporal dynamics in tip‐growing cells, providing a flexible alternative to manual image analysis and enabling studies of the molecular mechanisms underlying polarized growth.

## Graphical abstract

Tip Quantification enables quantitative analyses over versatile selections of regions of interest.

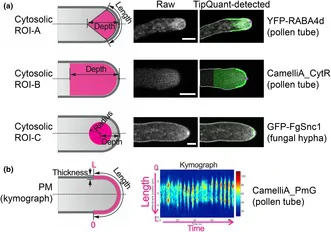

## Introduction

Cell polarity, a fundamental asymmetric property of the cell, is essential for its development and function, such as cell differentiation, movement, morphogenesis, and polar and directional growth. Cell polarity can be found in all cellular organisms. In all cases, the formation of cell polarity relies on the dynamic distribution and changes in the type and quantity of subcellular structures and molecules that are typically interconnected into a network at the polar site (Drubin & Nelson, 1996; Yang, 2008; Chiou *et al*., 2021). Understanding their spatial distribution over time (i.e. spatiotemporal dynamic) is critical for elucidating the mechanism and function for the formation of a specific cell polarity. Manual quantification of these dynamics is extremely time‐consuming, hindering progress in the field and limiting the integration of mathematical modeling into studies of cell polarity formation. Thus, computer‐aided robust automated analyses are needed to advance the field, but are challenging, especially in a cell system with moving or changing sites of polarity, which is quite common in moving, growing, and dividing cells.

Tip growth is an extreme form of polar growth characterized by a unidirectional expansion in the apical region of the cell and is found in many cell types throughout the eukaryotic kingdom, such as pollen tubes and root hairs in plants, hyphae/mycelia in fungi, and neuronal axons in animals (Yang, 2008; Qin & Yang, 2011). Pollen tube tip growth is required for growth through sporophytic tissues and for the delivery of sperm cells to the female gametophyte, both of which are essential for plant reproduction. Hyphal growth is essential for the colonization and invasion of fungi in the host tissues, while axon extension is necessary for neuronal development and function. Tip growth generates a highly elongated cell with a cylindrical shape and involves the expansion of the cell only in the dynamic apical region of the cell where membrane and extracellular matrix materials are inserted through exocytosis. In diverse tip‐growing cell models, this process is regulated by a dynamic network of signaling molecules and cytoskeletal structures controlled by Rho‐family GTPases. Hence, tip‐growing cells provide an excellent system for studying spatiotemporal coordination of dynamic cellular activities.

In particular, the pollen tube serves as an excellent model system for investigating such spatiotemporal coordination and the mechanisms underlying tip growth, because it can be conveniently manipulated and imaged *in vitro* and various genetic and molecular tools are available for this system. Pollen tube tip growth may oscillate rapidly with periods as short as 5–6 s (Guo *et al*., 2022). *In vivo* pollen tube growth is directional, guided by attractive signals secreted from the female tissues (Higashiyama *et al*., 2001; Okuda *et al*., 2009; Luo *et al*., 2017). As in other walled tip‐growing cell types, the growth of pollen tubes requires the expansion of the cell wall at the apex driven by internal turgor pressure, while the mechanical properties of the cell wall determine the morphology of the cell (Hepler *et al*., 2013). To achieve periodic and directional growth, the deposition of the cell surface materials is controlled precisely in space and time by a spatiotemporally coordinated signaling network that is centered on Rho‐like GTPase from plants (ROP) signaling and composed of multiple downstream pathways, feedforward and feedback loops, cytoskeletal dynamics, and influxes and effluxes of ions such as calcium (Yang, 2008; Hwang *et al*., 2010; Qin & Yang, 2011; Luo *et al*., 2017; Tian *et al*., 2019).

This network is characterized by its remarkable spatiotemporal dynamics (Cheung & Wu, 2008; Zonia, 2010; Guan *et al*., 2013). Spatially, it is highly polarized: dense populations of exocytic vesicles, supported by longitudinal actin filaments and an apical actin meshwork, move towards the extreme apex of pollen tubes, delivering cell membrane and cell wall components to the tip, while endocytosis retrieves excessive materials from the subapical or apical regions (Guo & Yang, 2020). Active ROP1 and its associated signaling components, localized to the apical membrane, play an essential role in maintaining cell polarity by regulating actin dynamics and exocytosis (Fu *et al*., 2001; Gu *et al*., 2005; Lee *et al*., 2008; Guo & Yang, 2020). Calcium ions also show a tip‐high gradient and are crucial for pollen tube growth by participating in multiple pathways including the ROP signaling (Pierson *et al*., 1996; Li *et al*., 1999; Yan *et al*., 2009; Guo *et al*., 2022). Temporally, this system is oscillatory and extremely dynamic: ROP1 activity, calcium concentration, and accumulation of vesicles at the cell tip exhibit rapid and correlated oscillations resulting in oscillating growth (Hwang *et al*., 2005; Lee *et al*., 2008; Yan *et al*., 2009). The mechanisms underlying the polar growth of other tip‐growing cells, such as pollen tubes, root hairs, and fungal hyphae, are evolutionarily conserved, and this growth can be mediated by different protein families or even different organizing structures, such as the Spitzenkörper, depending on the cell type and organism (Qin & Yang, 2011). Therefore, the study of polar growth in these cell models relies heavily upon the analysis of the distribution and dynamics of various molecules and structures, yielding large amounts of video image data that require quantification and interpretation, which is extremely time‐consuming by manual measurements.

Available algorithms or computational tools for analyzing tip‐growing cells have limited power and flexibility for analyzing the spatiotemporal dynamics of labeled molecular or structural components. For example, the CHUKNORRIS, an algorithm for detecting oscillations of ion fluxes as well as growth rate data, relies on a user‐generated kymograph; therefore, it has the element of user subjectivity and contains partial spatiotemporal information of molecular components along the central axis of the cell (Damineli *et al*., 2017). The Automated Stack Iterative Subtraction (ASIST) method (Ponvert *et al*., 2019) and the contour‐based coordinate normalization (CCN) method (Kang *et al*., 2023) have been successfully applied to analyze growth parameters of tip‐growing cells, such as pollen tubes, root hair, and zygotes. However, their analyses are only limited to growth rate and directionality while being unable to analyze spatiotemporal distribution of molecules and structural components. The TIGRMUM toolkit, detecting the tip of pollen tubes by weighting the ellipse method and morphological thinning (Burri *et al*., 2020), performs measurement of fluorescence intensity or ratio of two fluorescent channels over a fixed cytosolic region of interest (ROI) with the capability of analyzing the left and right half ROIs separately. AMEBaS (Automatic Midline Extraction and Background Subtraction of Ratiometric Fluorescence Time‐Lapses of Polarized Single Cells) generates kymographs along the midline of tip‐growing cells, lacking quantitative measurement over any user selectable ROIs (Badain *et al*., 2023). A more recently developed KymoTip allows users to analyze the growth behavior of plant tip‐growing cells and generate centerline kymographs for various fluorescent markers; however, it lacks functionality for quantifying intensity changes over time (Kang *et al*., 2026). We attempt to address this problem by initially developing a TipQAD algorithm to automatically quantify the spatiotemporal dynamics of fluorescently labeled molecules or structures on the plasma membrane (PM) and in the cytoplasm at the apex of tip‐growing cells (Tambo *et al*., 2020). In the TipQAD algorithm, the contour of a tip‐growing cell is first fitted with ellipses; the intersection of the PM and the major axis of the best‐fit ellipse is determined as the growing tip. While the TipQAD has provided better analyzing power over the previously developed methods, it suffers from several key issues: First, the tip determination highly depends on the shape of the growing cells; therefore, it is unable to reliably analyze cells that change shape dramatically, such as large changes in growth direction or cell diameter. Second, it only analyzes a fixed horseshoe‐shaped ROI in the cytoplasm, lacking the flexibility of ROI choices for quantitative analyses.

Here, we introduce TipQuant (Tip Quantification), a Python‐based automated tool designed for detecting tips and analyzing the spatiotemporal dynamics of fluorescently labeled molecules and structures on the PM and within the apical cytoplasm in tip‐growing cells. We have demonstrated the efficacy of TipQuant in correlating the spatiotemporal dynamics of two molecular events – ROP activity and PM Ca^2+^ influxes – within the same cell. Consequently, TipQuant proves to be an essential resource for quantitative analysis of spatiotemporal dynamics of signaling and structural components in tip‐growing cells. It also generates valuable quantitative data for mathematical and computational modeling studies aimed at a quantitative understanding of these dynamics.

## Materials and Methods

### Computing platform

The TipQuant code requires Python 3.11 or later. While it should work with newer versions, Python 3.11 is recommended. All necessary dependent packages, as listed in the *pyproject.toml* file, are automatically installed using Poetry, a Python package management tool. The code has been rigorously tested on macOS (a Macbook Pro M1 running macOS Ventura 13.4 and a MacBook Pro M5 running macOS Tahoe 26.3) and Windows (10 and 11) operating systems and has been shown to run smoothly.

### Video data

The video of *Arabidopsis thaliana* pollen tube expressing CRIB4‐GFP squeezing through the narrow‐gap region of a TipChip microfluid chamber was generated as described previously (Zhou *et al*., 2021).

The video of Arabidopsis pollen tube expressing CRIB4‐GFP turning toward the attractant AtLURE1.2 was kindly shared by Dr Nan Luo (Luo *et al*., 2017).

Videos of Arabidopsis pollen tubes expressing CamelliA_PmG (PM Ca^2+^ influx) and CamelliA_CytR (cytosolic Ca^2+^ level) are derived from the authors' previously published experimental observations (Guo *et al*., 2022).

Videos of *Fusarium graminearum* hyphae expressing GFP‐FgSnc1 were kindly provided by Dr Wenhui Zheng (Zheng *et al*., 2021).

Videos of Arabidopsis pollen tubes expressing YFP‐RABA4d were generated for this research. Briefly, pollen tubes expressing YFP‐RABA4d driven by the pollen‐specific promoter Lat52 (Szumlanski & Nielsen, 2009) were germinated on pollen germination medium and mounted for observation as previously described (Guo & Yang, 2020). The fluorescence emission of YFP‐RABA4d was collected between 525 and 600 nm while being excited by a 514 nm laser under a Leica SP5II confocal microscope equipped with a Leica HCX PL APO CS 40×/1.10 WATER UV objective lens (Leica, Germany).

Videos of Arabidopsis pollen tubes co‐expressing CRIB4‐GFP and CamelliA_PmR were generated as follows. A homozygous Arabidopsis line expressing both markers was produced through genetic crossing. Pollen tubes of the dual‐marker line were germinated, mounted as described above, and observed under a Leica STELLARIS FALCON FLIM Microscope equipped with a Leica HC PL APO CS2 40×/1.10 water‐immersion objective lens. The fluorescence emission of CRIB4‐GFP was collected between 498 and 570 nm while being excited by a 488 nm laser, and the emission of CamelliA_PmR was collected between 575 and 700 nm while being excited by a 561 nm laser. Both fluorophores were imaged sequentially to avoid fluorescence bleed‐through.

### Generation of the ground truth data

We used Fiji/ImageJ to measure and generate the ground truth (GT) data for the mean fluorescence intensity of PM signals, various cytosolic ROIs, and the PM kymograph.

#### Generation of the GT data of PM fluorescence intensity and kymograph

A 20 μm‐long PM region at the apical tip of CamelliA_PmG pollen tube is manually traced using the *segmented line* tool. The fluorescence intensity of pixels in the selected region is then read out using the *plot profile* function, with the line width set to 5 pixels. This process is repeated for every frame of the video to collect the full dataset of PM fluorescence intensity distributions over time. This collection is further processed using Pandas (McKinney, 2010) and scikit‐fda Python packages (Ramos‐Carreño *et al*., 2024) to generate the GT data for the PM kymograph. The mean PM fluorescence intensity over time is calculated as the average fluorescence intensity of the PM in each frame.

#### Generation of the GT data of fluorescence intensity in various cytosolic ROIs

Various cytosolic ROIs were manually traced using the *Polygon selections* tool in each time frame, followed by measuring mean fluorescence intensity.

### Realignment of GT PM kymograph with TipQuant‐generated kymograph

When comparing PM kymographs of CamelliA_PmG pollen tubes generated by manual measurement (GT) and TipQuant (TQ), an important challenge in comparing the two intensities $I^{\mathrm{GT}}\left(x,t\right)$ and $I^{\mathrm{TQ}}\left(x^{\prime },t\right)$ is that they are not defined on the same spatial coordinate systems x and $x^{\prime }$. Indeed, in both cases, the tip detection algorithms produce two different plane curves $C^{\mathrm{GT}}=C^{\mathrm{GT}}\left(x\right)$ and $C^{\mathrm{TQ}}=C^{\mathrm{TQ}}\left(x^{\prime }\right)$ that can be parametrized by their respective arc lengths $x,x^{\prime }$ (with total length $L^{\mathrm{GT}},L^{\mathrm{TQ}}$). The coordinate x represents the distance along the curve $C^{\mathrm{GT}}$ between the point at location x and the left boundary $C^{\mathrm{GT}}\left(0\right)$. The intensities $I^{\mathrm{GT}},I^{\mathrm{TQ}}$ are functions defined on $C^{\mathrm{GT}}$ and $C^{\mathrm{TQ}}$ and we need to align them to ensure a fair comparison of the intensities. This requires finding a way to match every point $C^{\mathrm{GT}}\left(x\right)$ to a unique point $C^{\mathrm{TQ}}\left(x^{\prime }\right)$ by determining the best function h. The function h establishes a one‐to‐one correspondence between the points of each tip: $h\left(C^{\mathrm{GT}}\left(x\right)\right)=C^{\mathrm{TQ}}\left(x^{\prime }\right)$ (and we simply write $h\left(x\right)=x^{\prime }$). This correspondence $h:C^{\mathrm{GT}}\to C^{\mathrm{TQ}}$ is necessary to account for variations in the localization of the tip membrane. The simplest alignment corresponds to the case where the curves differ only by a translation by a factor c, and the two curves are matched with the function $h\left(x\right)=x+c$. The translation c is unknown and is selected to maximize the similarity between $I^{\mathrm{GT}}\left(x,t\right)$ and $I^{\mathrm{TQ}}\left(x+c,t\right)$ (assuming that $L^{\mathrm{GT}}<L^{\mathrm{TQ}}$): the intensity functions must represent the same quantity.

In general, as the detected contours might involve a nonlinear transformation from one to the other, we need to consider more complex transformation $h\left(x\right)$ for aligning $I^{\mathrm{GT}}\left(x,t\right)$ and $I^{\mathrm{TQ}}\left(h\left(x\right),t\right)$. The computation of an optimal alignment (warping) function $h\left(x\right)$ can be achieved by minimizing the distance between the two functions $I^{\mathrm{GT}}\left(\cdot ,t\right)$ and $I^{\mathrm{TQ}}\left(\cdot ,t\right)$. We consider the so‐called Fisher‐Rao distance for computing the best alignment, as it allows detecting similar shapes effectively. It is considered one of the reference methods for alignment or registration of curves because it exhibits desirable theoretical properties like invariance to parametrization and provides good results when used on real cases (Srivastava & Klassen, 2016). This distance involves the derivatives of the functions $I^{\mathrm{GT}}\left(\cdot ,t\right)$ and $I^{\mathrm{TQ}}\left(\cdot ,t\right)$ (since the derivative characterizes the peaks and valleys of $I^{\mathrm{TQ}}\left(\cdot ,t\right)$ more than $I^{\mathrm{TQ}}\left(\cdot ,t\right)$ itself), and we have used the implementation proposed in the Python Open Source Package scikit‐fda (https://github.com/GAA‐UAM/scikit‐fda).

### Comparison of GT and TipQuant‐generated fluorescence intensity measurements

We use *corr* function in matlab R2022a to calculate the Pearson's linear correlation coefficient between the GT and TipQuant‐generated fluorescence intensity vs time data arrays.

## Results

### Algorithm development

**Fig. 1:**
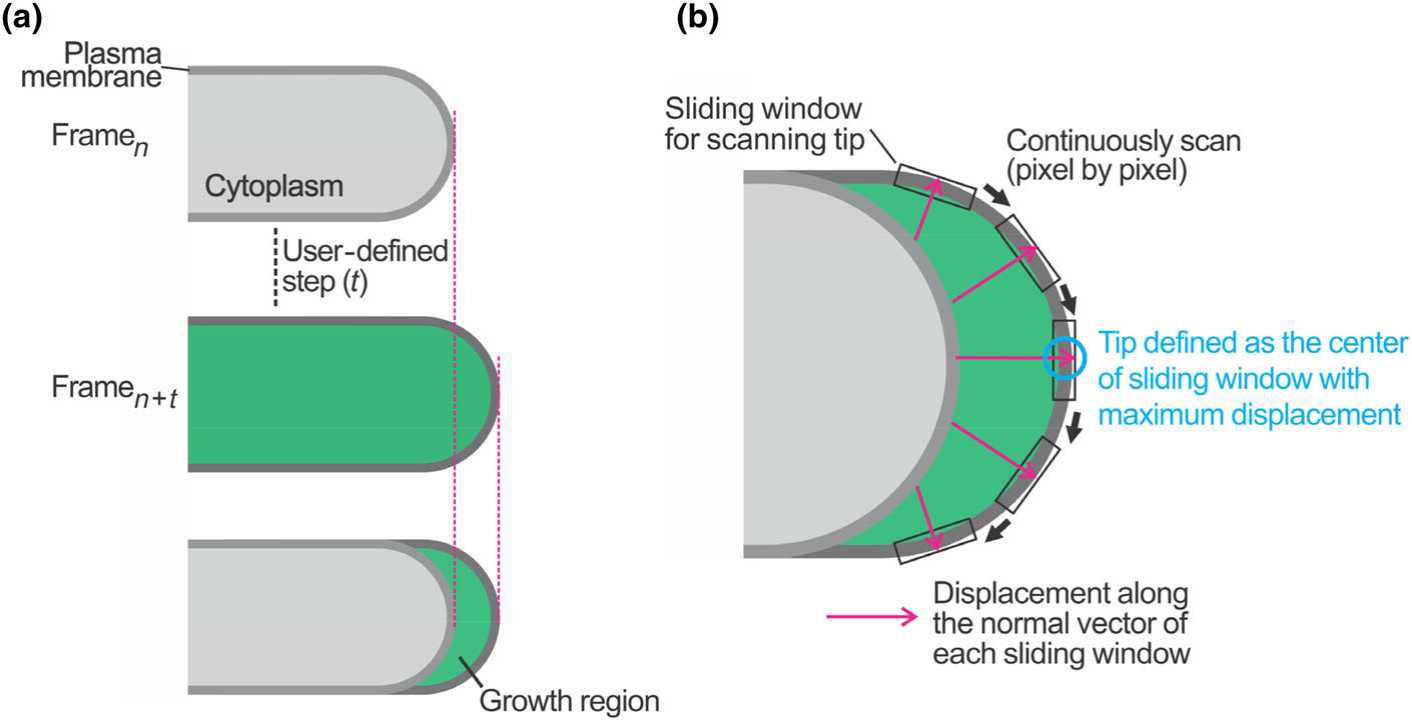
Simplified schematic depicting the process of tip detection. The tip detection involves two steps: growth region detection (a) and tip determination on the plasma membrane (PM) (b). In the first step (a), the growth region is identified by the spatial dislocation of cell contour between two frames separated by a user‐defined step (Frame_*n*_ vs Frame_*n*+*t*_). In the next step (b), a sliding window (of user‐defined pixel numbers, depicted by the black rectangle along the PM) moves along the contour pixel by pixel; a normal vector is then computed for each window after calculating its curvature. Subsequently, the displacement of each sliding window along its normal vector (illustrated by the magenta arrow) is determined, and the center of the sliding window with the maximum displacement is defined as the tip.

Central to the automated analyses of tip‐growing cells is robust detection of growing tips, especially when pollen tubes have complicated growth patterns such as turning and zigzagging. Tip growth is defined by directional deposition of membranes and/or cell wall materials, such that a directional expansion of the cell is accompanied by an observable morphological change. Therefore, we developed a tip detecting algorithm which defines the growing tip as the site with maximum expansion. Specifically, for a given video series, the algorithm first performs contour segmentation of a tip‐growing cell on all frames (see Supporting Information Methods S1 for details of contour segmentation), then detects the growing region by identifying maximum spatial dislocation of cell contour between two frames parted with a user‐defined step (Frame_*n*_ vs Frame_*n*+*t*_; Fig. 1a). During the tip detection, we create a sliding window (in user‐defined pixel numbers) moving along the expanded contour pixel by pixel; we then calculate the curvature of the window and create a normal vector for each sliding window. Finally, we calculate the displacement of each sliding window along its normal vector and define the center of the sliding window with the maximum displacement as the tip (Fig. 1b, also see Methods S2 for details of tip detection). Based on the algorithm‐recognized contour, including spatial coordinates of contour and tip position, we further developed codes for measuring mean fluorescence intensity on user‐defined PM and cytosolic ROI, cell growth by area expansion, as well as PM kymograph (see detailed coding information in Methods S3).

### TipQuant robustly segments and detects growing tips

**Fig. 2:**
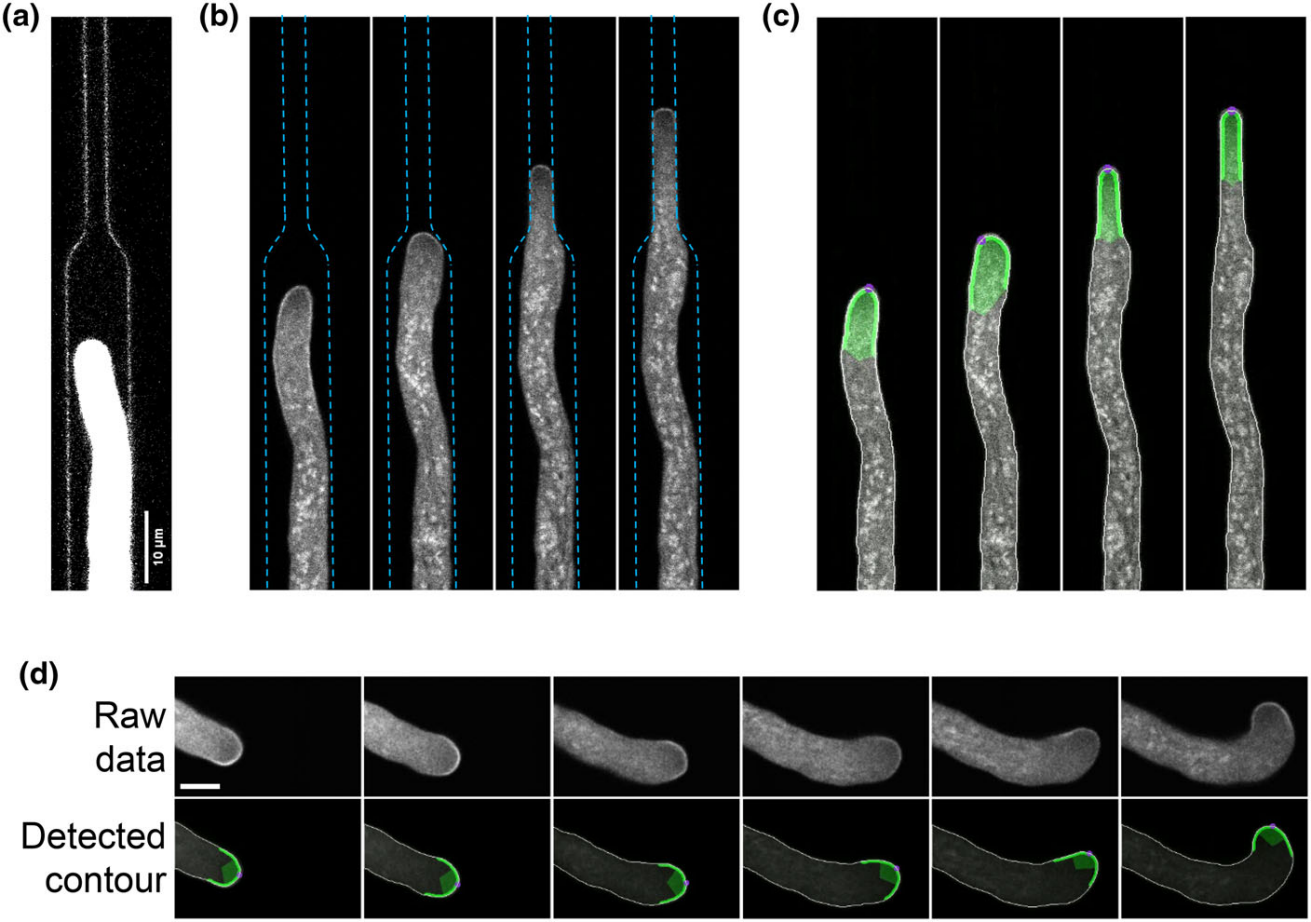
Dynamic segmentation and tip detection of tip‐growing cells with substantial morphological changes. (a–c) Tip detection of an Arabidopsis CRIB4‐GFP pollen tube growing on a TipChip microfluidic channel. The channel comprises a wide part and a narrow part (shown in (a) by enhanced image contrast), and the pollen tube growing in the wide channel is forced to squeeze into the narrow channel (raw image shown in (b) with the contour of the channel highlighted by the blue dotted line). The output of TipQuant‐detected pollen tube is shown in (c). Bar, 10 μm. (d) Tip detection of an Arabidopsis CRIB4‐GFP pollen tube exhibiting frequent turning behaviors in response to manually applied pollen tube attractant AtLURE1. The upper panel shows raw images, and the lower panel shows the TipQuant‐detected pollen tube. Bar, 5 μm. In all TipQuant‐detected images, the tip is marked with purple circle, the PM, and cytosolic regions at the tip are highlighted with brighter green lines and lighter green shades, respectively.

To validate the performance of our tip detection algorithm, we challenged the code with two types of *Arabidopsis thaliana* pollen tubes with complicated growth patterns: pollen tubes forced to become narrower when squeezing through a narrow microfluid channel (Zhou *et al*., 2021) and pollen tubes turning to a female attraction signal AtLURE1 (Luo *et al*., 2017). In the first case, an Arabidopsis pollen tube expressing the ROP activity reporter CRIB4‐GFP is growing from a wide channel (9 μm in width) to a narrow channel (4 μm in width) inside a TipChip microfluid device (Zhou *et al*., 2021; Fig. 2a). With a typical diameter of 6 μm, the pollen tube was forced to 4 μm to squeeze through the narrow channel (Fig. 2b). Nevertheless, our code still robustly segregates and detects the growing tip throughout the shape‐changing process (Fig. 2c). In the second case, we ask the code to track the growing tip of an Arabidopsis pollen tube turning toward the AtLURE1 attraction peptides (Fig. 2d). Although the pollen tube has made several turns and grown into different focal planes compared to the first few frames, our code still manages to track the growing tip (Fig. 2d). We also have successfully segregated and tracked the growing tip of fungal hyphae (see later results), indicating the wide applicability of our code to various tip‐growing cells. These results demonstrate the robustness of our code for segmentation and tip detection in tip‐growing cells regardless of their growing behavior.

### Quantitative measurement on user‐defined ROIs and PM kymograph generation

Quantifying spatiotemporal changes in molecule distribution is crucial for understanding the mechanisms regulating tip growth. As previously mentioned, current methods for analyzing tip‐growing cells either lack the capability or have limited power to generate kymographs and perform quantitative measurements over ROIs. TIGRMUM, AMEBaS, and KymoTip are tools capable of generating kymographs along the central line of tip‐growing cells and measuring fixed ROIs at the tip (Burri *et al*., 2020; Badain *et al*., 2023; Kang *et al*., 2026).

By contrast, our TipQuant code offers flexible options for selecting ROIs, with three different user‐adjustable shapes of ROIs within the cytoplasm and on the PM for quantitatively analyzing mean fluorescence intensity. Additionally, TipQuant can analyze fluorescence intensity distribution on user‐defined PM regions and generate a 2D kymograph of the spatiotemporal distribution of labeled molecular or structural components on the PM.

#### Quantitative measurement on user‐defined cytosolic ROIs

**Fig. 3:**
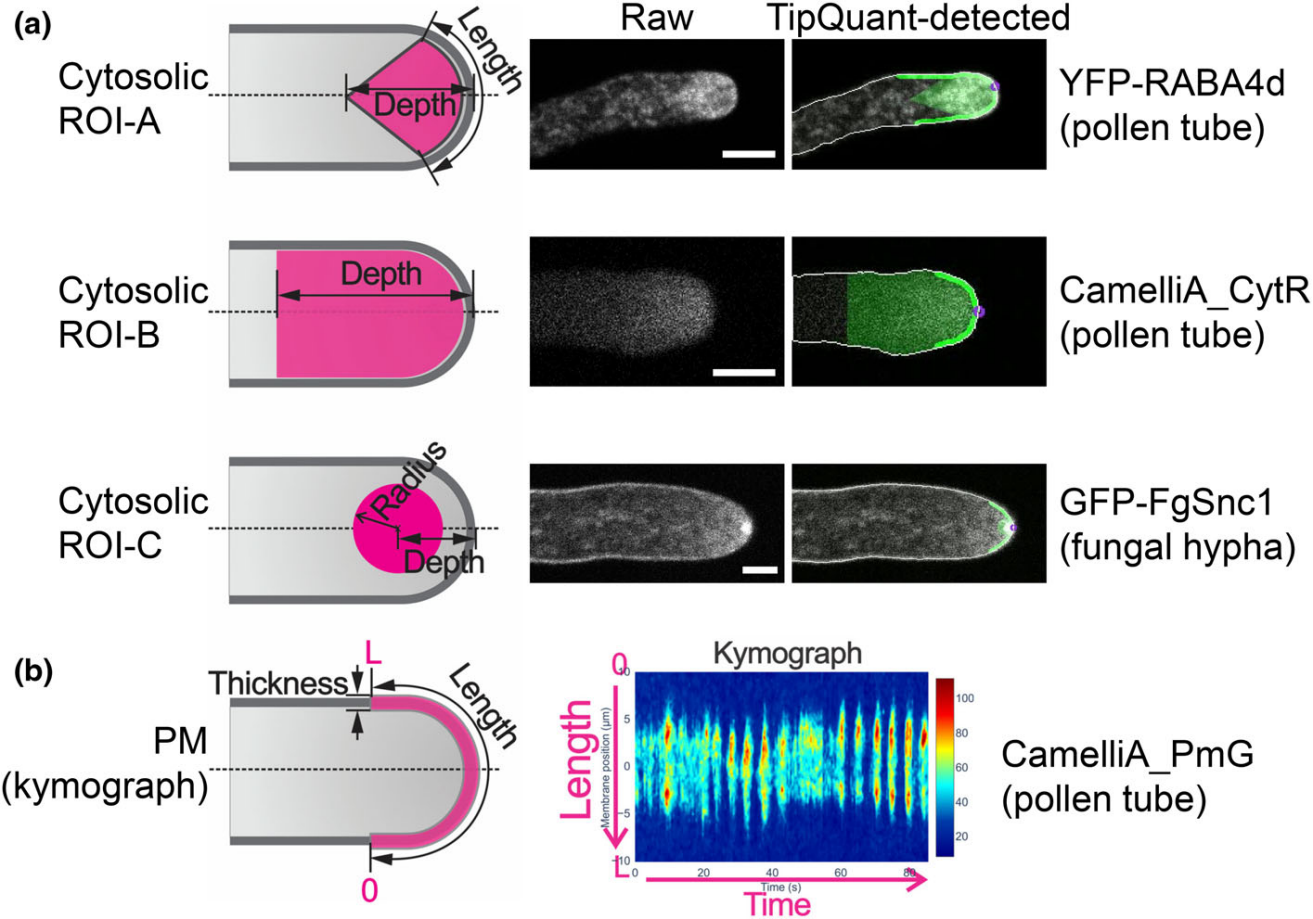
TipQuant enables quantitative analyses over versatile selections of regions of interest (ROIs). (a) The code allows measuring the mean fluorescence intensity of cytosolic ROIs of a V‐cone‐shaped region (ROI‐A), whole cytosolic region from tip to a user‐defined depth (ROI‐B), and circular region within the cytoplasm (ROI‐C). All three types of cytosolic ROIs have user‐adjustable parameters to fit the subcellular patterns of proteins or dyes of interest (left column). The middle and right columns show the representative raw images of the tip‐growing cells (middle column) and the TipQuant‐detected results (right column) with light green shades highlighting the cytosolic region for measurement. Bars, 5 μm. (b) Mean fluorescence intensity of a plasma membrane (PM) region spanning evenly from the tip with a user‐defined length and thickness (left panel) along with a kymograph generated by TipQuant (right panel). Videos of Arabidopsis pollen tubes expressing YFP‐RABA4d, CamelliA_CytR, and CamelliA_PmG were used for TipQuant analysis of cytosolic ROI‐A, ROI‐B, and PM kymograph, respectively. A video of a *Fusarium graminearum* hypha expressing GFP‐FgSnc1 was used for cytosolic ROI‐C analysis.

Quantifying cellular and signaling components, which often have distinct subcellular localizations, requires highly adaptable ROIs to accommodate their unique spatial distribution patterns. To address this need, our TipQuant code offers three types of cytosolic ROIs for measuring signal intensities: a V‐cone‐shaped region (ROI‐A), a whole cytosolic region from tip to shank (ROI‐B), and a circular region within the cytoplasm (ROI‐C; Fig. 3a). These ROIs are adjustable in both position and size, making them suitable for analyzing a wide range of proteins with diverse subcellular localizations. Specifically, the V‐cone‐shaped ROI‐A is ideal for proteins involved in polar secretion, the whole cytosolic ROI‐B is suitable for most cytosolic proteins and reporters of various ions or signaling molecules, and the circular ROI‐C within the cytosolic tip is designed for specific subregions, such as the characteristic Spitzenkörper in fungal hyphae.

#### Quantitative measurement on user‐defined PM ROI and PM kymograph generation

The PM, as the primary interface between the cell and its environment, hosts numerous signaling proteins, including receptors, ion transporters, GTPases, etc., which orchestrate cellular activity in response to internal and environmental cues. These signaling events often trigger changes in ion influxes, enrichment/depletion of PM‐localized proteins, or the formation of protein subdomains on the PM. To better understand these cellular processes, the TipQuant code includes functions for spatial and temporal quantification on the PM by providing measurements of mean fluorescence intensity and generating kymograph. By accurately tracking the extreme tip of the cell, the TipQuant code allows users to measure fluorescence intensity distribution along a user‐defined PM region centered around the apex, which is then converted into kymograph slices for each time frame. A complete PM kymograph is generated by aggregating these slices, providing a dynamic view of the PM activity over time, together with the corresponding mean PM fluorescence intensity (Fig. 3b).

### TipQuant‐generated quantitative data faithfully represent the GT

**Fig. 4:**
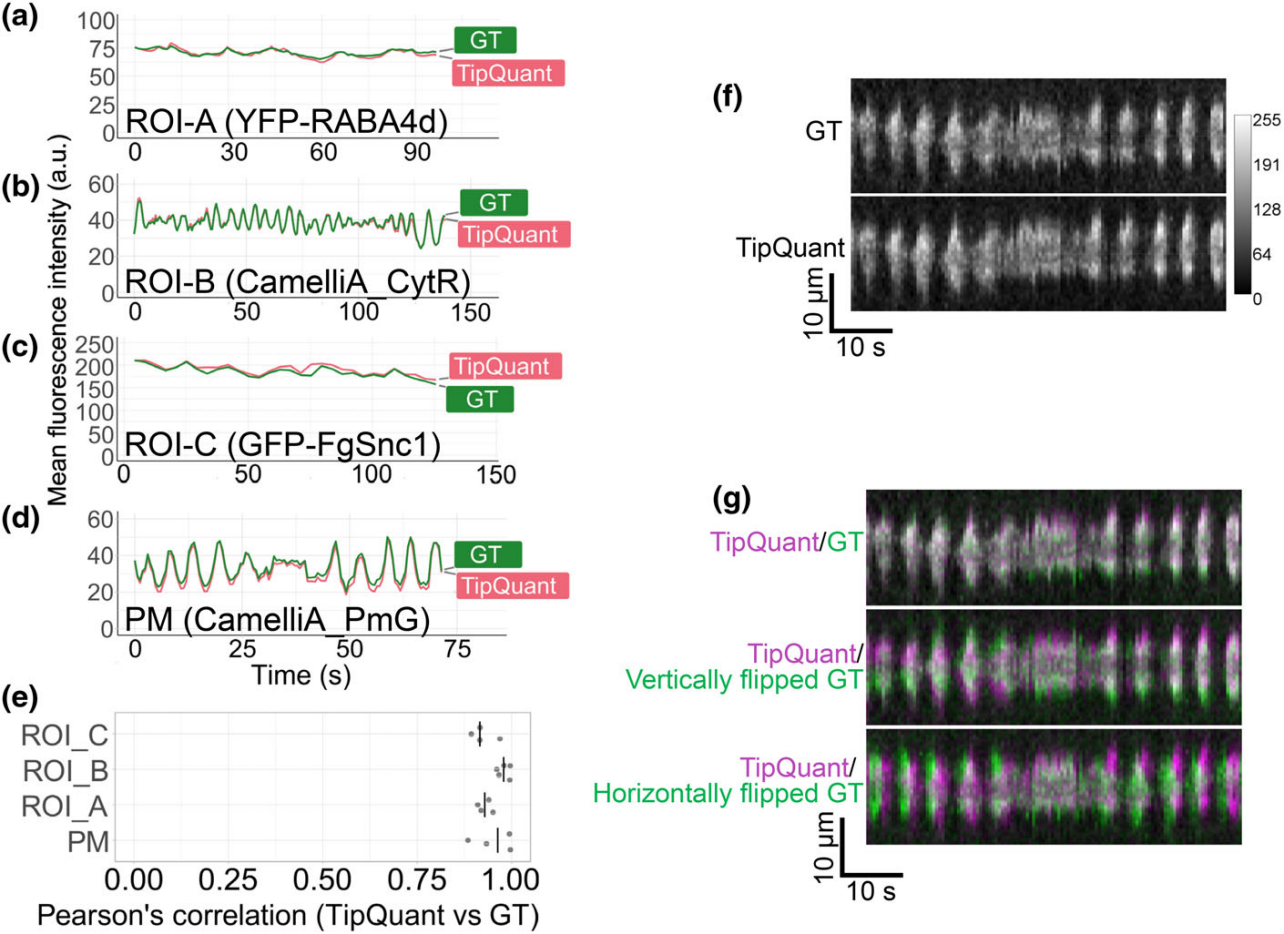
Validation of TipQuant‐generated quantitative data and kymograph. (a–d) Representative plots comparing ground truth (GT) and TipQuant‐generated mean fluorescence intensity changes over time in cytosolic ROI‐A (a), ROI‐B (b), ROI‐C (c), and plasma membrane (PM) region (d). The corresponding video data were obtained from Arabidopsis pollen tubes expressing YFP‐RABA4d (a), CamelliA_CytR (b), and CamelliA_PmG (d), and from *Fusarium graminearum* hyphae expressing GFP‐FgSnc1 (c). (e) Plot of Pearson's correlation coefficients between GT and TipQuant‐generated data for the ROIs measured above. Each dot represents an independent sample; vertical bars indicate mean values. (f–g) Validation of the PM kymograph of CamelliA_PmG generated by manual measurement (GT) and TipQuant (f). Composite images of magenta kymograph generated by TipQuant superimposed with green‐colored GT kymograph (g, top panel) or vertically flipped and horizontally flipped GT kymograph (g, middle and lower panels, respectively). The white color indicates perfect match between the two compared kymographs, whereas the more distinct nonoverlapping magenta and green colors, the poorer the match.

The correctness and coherence of the TipQuant methodology are evaluated by comparing the TipQuant output with manually measured data using ImageJ, which serves as the GT. The mean fluorescence intensity measurements over time across three distinct cytosolic ROIs and PM region are compared with their respective GT data (Fig. 4a–d). All comparisons demonstrate a high level of consistency, as indicated by the strong Pearson's correlation (Fig. 4e).

We also compared the TipQuant‐generated PM kymograph with manually curated data. The manually generated kymograph was created by vertically stacking intensity distributions measured over a 20 μm PM region flanking the tip apex at each time frame. Due to human subjectivity, the determination of the tip apex was arbitrary and not consistent with the apex determined by TipQuant. Therefore, a direct comparison between the two kymographs is not feasible. To address this, we first realigned the manually generated kymograph with the TipQuant‐generated kymograph (Fig. 4f, lower panel) by shifting the Y‐coordinate of each PM slice to align with the TipQuant data (Fig. 4f, see ‘Realignment of GT PM kymograph with TipQuant‐generated kymograph’ in the Materials and Methods section). We then compared the two kymographs by superimposing the color‐encoded kymographs using imagej software, with magenta and green indicating the TipQuant‐generated and manually curated kymographs, respectively; matched patterns appear white when superimposed (Fig. 4g). To further validate the accuracy of the TipQuant‐generated kymograph, we performed the same superimposition process between a vertically or horizontally flipped manually curated kymograph and the TipQuant‐generated kymograph. The results indicate a high degree of correspondence only between the TipQuant‐generated kymograph and the manually curated data (Fig. 4g).

### TipQuant revealed temporal relationship between oscillations of PM ROP activity and Ca^2+^ influx

**Fig. 5:**
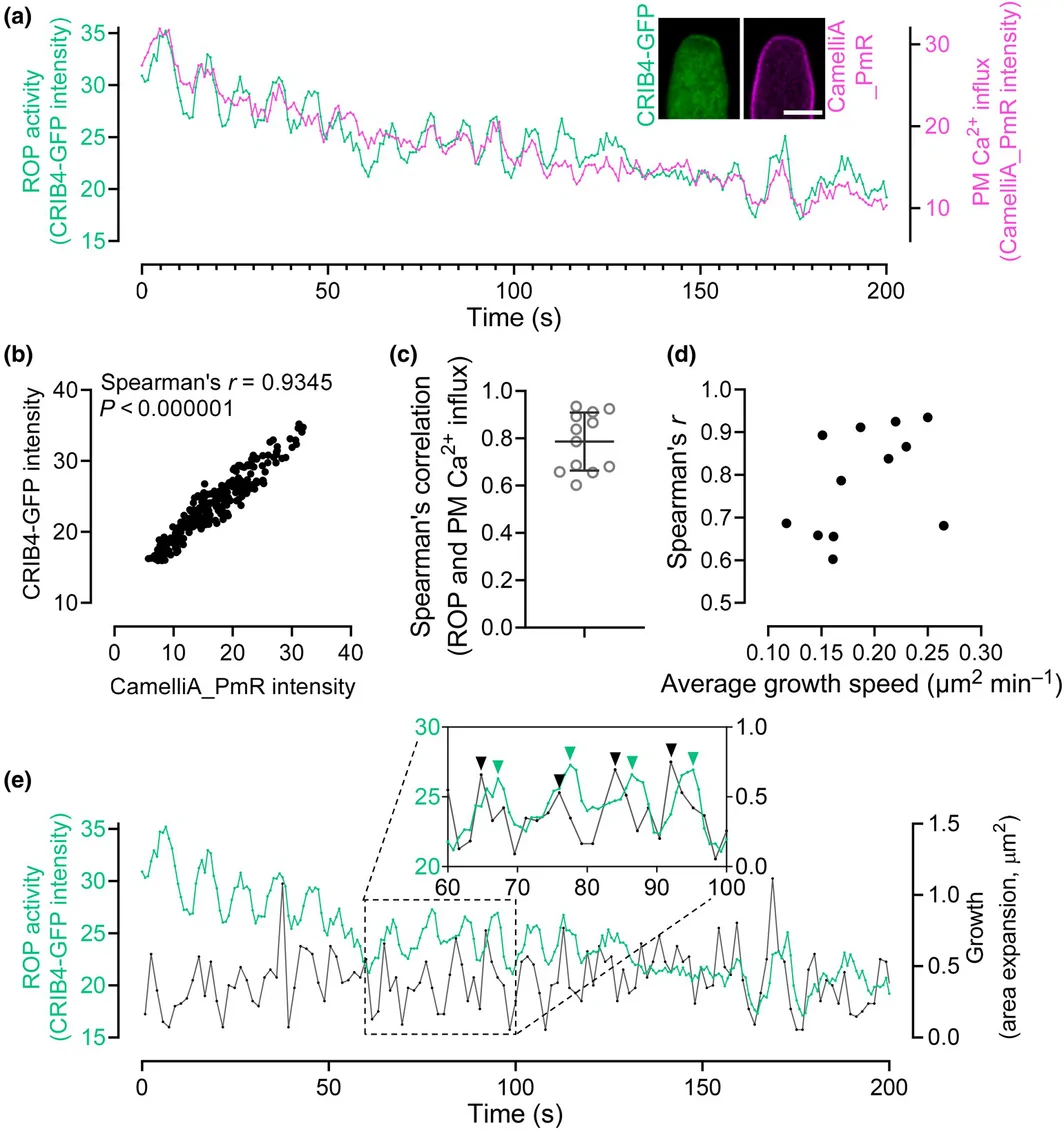
Simultaneous analysis of Rho‐like GTPase from plants (ROP) activity and plasma membrane (PM) Ca^2+^ influx in Arabidopsis pollen tubes. (a) Time‐course plot showing mean PM fluorescence intensity changes for the ROP activity reporter CRIB4‐GFP and the PM Ca^2+^ influx sensor CamelliA_PmR. Insets show representative images of the pollen tube expressing CRIB4‐GFP (left) and CamelliA_PmR (right). (b) Scatter plot of paired CRIB4‐GFP and CamelliA_PmR intensity values at each time point, with Spearman's correlation coefficient (*r*) and *P*‐value indicated at the top. (c) Scatter plot of the Spearman's correlation coefficients (*r*) between CRIB4‐GFP and CamelliA_PmR intensities from videos of 12 individual pollen tubes. Error bar represents the mean ± SD. (d) Scatter plot of Spearman's correlation coefficient (*r*) vs average growth rate for 12 individual pollen tubes. (e) Time‐course plot of ROP activity (CRIB4‐GFP fluorescence intensity at the PM) and pollen tube growth measured by area expansion. The inset highlights the temporal relationship between peaks of ROP activity (green arrowheads) and growth (black arrowheads). Bar, 5 μm in (a).

After validating the fidelity of the TipQuant‐generated measurements, we examined the potential of TipQuant to improve our understanding of signaling events in cellular processes. Using the ROP GTPase activity reporter CRIB4‐GFP (Luo *et al*., 2017) and the PM Ca^2+^ influx reporter CamelliA_PmR (Guo *et al*., 2022) in Arabidopsis pollen tubes, we quantified temporal changes in these two key signaling components at the PM with TipQuant. Fig. 5(a) shows a representative pollen tube expressing both reporters, together with TipQuant‐based quantification of the PM ROP activity and Ca^2+^ influx using the mean fluorescence intensity at the PM as readout. Spearman's correlation analysis of video data from 12 independent pollen tubes revealed a strong positive correlation between oscillations in ROP activity and PM Ca^2+^ influx (Figs 5b,c, S1). This strong positive correlation contrasts with the previously proposed *c*. 130° lag of cytosolic Ca^2+^ oscillations behind ROP activity (Hwang *et al*., 2005), although that conclusion was inferred from observations of pollen tubes treated with Ca^2+^ channel blocker. By directly monitoring ROP activity and Ca^2+^ influx in the same cell, our analyses provide quantitative datasets of their spatiotemporal relationship and establish a foundation for future study of the feedback regulation between ROP and Ca^2+^ signaling using experimental and mathematical modeling approaches (Tian *et al*., 2019; Xiao *et al*., 2022).

Notably, the strength of the correlation, as measured by Spearman's coefficient (*r*), varied among individual pollen tubes, ranging from 0.6021 to 0.9345. We therefore ask whether this variation is associated with pollen tube growth rate. However, we did not detect any correlation between average growth speed and the Spearman's coefficient (*r*) (Fig. 5d). Using the growth measurement function of TipQuant, we further found that ROP activity oscillations consistently lagged behind growth oscillations (Figs 5e, S2). In addition, pollen tube elongation was reduced when ROP activity reached its peak during each oscillatory cycle (Figs 5e, S2), consistent with our previous finding that ROP activity was negatively correlated with pollen tube elongation (Zhou *et al*., 2021).

## Discussion

We have developed and extensively tested the TipQuant code, demonstrating its ability to automatically and accurately analyze the spatiotemporal distribution of key molecular and structural components in tip‐growing cells in an unbiased manner. It has shown exceptional capability in analyzing various user‐defined ROIs, thereby facilitating the discovery of hidden information within existing confocal microscopy imaging datasets.

Our code detects growing tips by identifying the site with maximum spatial displacement, rather than relying on ellipse fitting as applied in several other reported codes (Burri *et al*., 2020; Tambo *et al*., 2020). Moreover, we offer users greater flexibility in selecting desired ROIs for confocal microscopy data analysis compared to other existing codes. Consequently, our code offers several distinct advantages over existing alternatives, as discussed below.

First, our TipQuant code can effectively process tip‐growing cells with complex growth behaviors, as demonstrated by its ability to segment and track pollen tubes that undergo dramatic changes in diameter when growing into the microfluidic chamber and changes in growth direction when navigating multiple turns toward the AtLURE peptide. However, we did notice that tip detection becomes less robust for cells that turn dramatically, such as turning more than 90° or making a U‐turn. Second, our code offers maximum flexibility by allowing users to define ROIs on both the PM and in cytosolic regions for unbiased and automated data analysis. Last, our code generates an advanced dataset by providing kymograph data along the PM, the primary interface for signaling transduction with the external environment, compared to traditional kymograph, which only displays data along the central axis.

In addition, we tested our code on two different types of tip‐growing cells – pollen tubes and fungal hyphae – using images acquired on major commercial confocal microscopy systems from Leica, Nikon, and Zeiss, with Nyquist sampling rates between 0.5× and 2×. These tests demonstrate the code's promising applicability to various tip‐growing cells and its compatibility with a wide range of confocal microscopy platforms.

Despite these advantages, the TipQuant algorithm is not applicable to nongrowing cells, as its core detection mechanism relies on cell contour expansion between frames. However, quantitative measurements of nongrowing cells can be readily performed using fixed ROIs in widely used image analysis software such as ImageJ (Schneider *et al*., 2012). Although uncommon, some tip‐growing cells produce multiple tips (Jones *et al*., 2002; Wudick *et al*., 2018). Currently, TipQuant cannot process videos featuring multiple growing tips; however, users can overcome this by cropping the video to isolate a single growing tip before analysis. Additionally, TipQuant‐generated quantitative data inherently lack information from the last few frames (determined by the user‐defined step ‘*N*’ for cell expansion detection) due to the absence of reference frames for tip detection. Last, TipQuant currently works only with confocal imaging data, as it is not programmed to handle out‐of‐focus blur at cell contour boundaries in epifluorescence microscopy images.

## Competing interests

None declared.

## Author contributions

J.G., Z.Y and X.C. conceptualized the research; JLG, RR and NB developed the TipQuant code with input from JG, prepared the software‐related sections of the manuscript and maintained the TipQuant GitHub repository. JG conducted experimental observations, generated ground truth data and wrote the original draft; AZ and XZ acquired videos of the Arabidopsis pollen tubes co‐expressing CRIB4‐GFP and CamelliA_PmR as well as Arabidopsis pollen tube passing through the microfluidic channel; ZY and XC acquired funding; NB, ZY and XC supervised the research and revised the manuscript; all authors have read and approved the manuscript. JG and JLG contributed equally to this work.

## Disclaimer

The New Phytologist Foundation remains neutral with regard to jurisdictional claims in maps and in any institutional affiliations.

## Supporting information


**Fig. S1** Simultaneous analysis of Rho‐like GTPase from plants activity and plasma membrane Ca^2+^ influx in Arabidopsis pollen tubes (additional data related to Fig. 5a–d).
**Fig. S2** Temporal relationship between Rho‐like GTPase from plants activity and growth oscillations in Arabidopsis pollen tubes (additional data related to Fig. 5e).
**Methods S1** Contour segmentation.
**Methods S2** Tip detection.
**Methods S3** Measurement of mean fluorescence intensity and generation of plasma membrane kymograph.
Please note: Wiley is not responsible for the content or functionality of any Supporting Information supplied by the authors. Any queries (other than missing material) should be directed to the *New Phytologist* Central Office.

## Data Availability

All raw video data used in the manuscript are available on Dryad through doi: 10.5061/dryad.dr7sqvbd2. The TipQuant code is accessible through Github: https://github.com/julianlegouic/TipQuant/.
